# Expressive writing combined with digital cognitive therapy in patients with schizophrenia: a narrative review of efficacy, linguistic phenotypes, and adherence modulators

**DOI:** 10.3389/fpsyt.2026.1804706

**Published:** 2026-06-04

**Authors:** Haimei Wen, Yafang Liu, Yu Wang, Yuli Zhang

**Affiliations:** 1 Department of Nursing, Nanjing You’an Hospital, Nanjing, Jiangsu, China; 2 Department of General Psychiatry II, Nanjing You’an Hospital, Nanjing, Jiangsu, China; 3 Department of Mental Rehabilitation III, Nanjing You’an Hospital, Nanjing, Jiangsu, China

**Keywords:** adherence, digital cognitive therapy, expressive writing, linguistic phenotype, schizophrenia

## Abstract

Schizophrenia involves emotional and cognitive deficits that are often unresponsive to medication, emphasizing the need for effective psychosocial rehabilitation. This narrative review and conceptual perspective explores the potential integration of expressive writing (EW) and digital cognitive therapy (DCT) as a proposed dual approach to support emotional regulation and cognitive recovery. EW can facilitate emotional processing and motivation, while digital therapy may improve attention, memory, and executive function through structured and interactive training. Their proposed combination could promote engagement, strengthen self-efficacy, and support long-term rehabilitation. Linguistic analysis using natural language processing (NLP) could offer objective indicators of emotional and cognitive change, potentially enabling personalized and adaptive intervention. Overall, the proposed integration model may provide a practical and broadly implementable framework for improving functional prognosis and promoting recovery in schizophrenia, but empirical research is needed to determine its effectiveness, scalability, or clinical applicability, among other aspects.

## 1 Introduction

Schizophrenia is a complex and chronic psychiatric disorder characterized by profound disturbances in thought, emotion, and behavior (1). In addition to positive symptoms such as hallucinations and delusions, patients often suffer from negative symptoms including emotional blunting, anhedonia, social withdrawal, and loss of motivation (2). Cognitive impairments involving attention, memory, and executive function are also common and significantly contribute to poor functional outcomes. These negative and cognitive symptoms are often resistant to pharmacological treatment and represent major obstacles to long-term recovery and social reintegration. Although antipsychotic medications remain the primary therapeutic approach, they mainly target positive symptoms and show limited efficacy in improving emotional and cognitive dysfunction. As a result, many patients continue to experience marked social disability and reduced quality of life despite adequate pharmacotherapy.

The limited efficacy of pharmacological treatment in addressing negative and cognitive symptoms highlights the need for effective non-drug interventions. Cognitive behavioral therapy (CBT) and psychosocial rehabilitation are currently the most widely used psychological approaches and have demonstrated benefits in improving insight, coping ability, and social functioning (3, 4). However, these interventions require intensive therapist involvement, substantial time investment, and consistent patient engagement, which restrict their scalability in real-world clinical settings. Furthermore, the abstract and cognitively demanding nature of CBT often poses challenges for patients with low motivation and cognitive deficits, resulting in inconsistent therapeutic outcomes (5). Therefore, developing accessible, cost-effective, and emotionally engaging interventions is essential for improving rehabilitation and functional recovery in schizophrenia.

In recent years, expressive writing (EW) and digital cognitive therapy (DCT) have emerged as promising complementary strategies for psychiatric rehabilitation (6). EW, which encourages individuals to articulate emotions and thoughts through structured writing tasks, facilitates emotional regulation, cognitive reappraisal, and self-integration (7). Studies in various psychiatric and medical populations suggest that EW can reduce psychological distress, enhance self-understanding, and promote adaptive coping. In schizophrenia, writing-based interventions may help patients reconstruct personal meaning, alleviate internalized stigma, and enhance motivation for social participation (8).

DCT, encompassing computer-based cognitive training, mobile applications, and virtual or augmented reality programs, provides an innovative and scalable platform for cognitive and behavioral interventions; here, “scalable” refers to interventions that can be effectively expanded to larger populations without proportional increases in resources or costs. Clinical studies have shown that DCT can improve attention, working memory, and executive function, while its interactive and self-paced design increases accessibility and patient engagement (9). Nevertheless, adherence remains a critical determinant of effectiveness, emphasizing the need to integrate emotional motivation with cognitive rehabilitation to achieve sustained improvement.

The integration of EW and DCT represents a proposed conceptual model that may target both emotional and cognitive domains of schizophrenia rehabilitation. EW may theoretically enhance intrinsic motivation and emotional engagement, thereby potentially facilitating participation in digital cognitive training. Meanwhile, digital platforms can provide structured feedback and objective monitoring, strengthening the continuity and efficacy of intervention (10, 11). Moreover, advances in natural language processing (NLP) have enabled the identification of specific linguistic phenotypes in schizophrenia, such as reduced lexical diversity and increased first-person pronoun usage, which are associated with symptom severity and may inform therapeutic monitoring.

This narrative review aims to summarize selected evidence on EW and DCT in schizophrenia and to propose a conceptual integrative framework combining emotional and cognitive pathways, and to explore how linguistic phenotypes and adherence mechanisms could inform the development of individualized, scalable rehabilitation strategies. The central premise of combining EW and DCT is presented as a hypothesis to be tested, acknowledging the current lack of direct evidence for the combined intervention in this population.

## 2 Methods

This brief section clarifies the non-systematic, narrative nature of the review. It synthesizes selected literature to provide a conceptual overview and discussion of key themes related to EW, DCT, linguistic phenotypes, and adherence in schizophrenia. It acknowledges that a formal systematic search strategy, inclusion/exclusion criteria, and quality appraisal were not conducted, as the aim is to integrate insights from diverse sources (including clinical trials, observational studies, and theoretical papers) to build a cohesive narrative on the topic. The cited literature was selected based on its relevance to the core themes of efficacy, linguistic analysis, and adherence in the context of the combined intervention.

### 2.1 Expressive writing in schizophrenia

#### 2.1.1 Theoretical foundations

EW is a structured intervention that guides individuals to describe their thoughts and emotions surrounding meaningful or distressing experiences through written expression. Its therapeutic effect is primarily achieved through three interconnected mechanisms: emotional release, meaning construction, and cognitive reappraisal (12). Emotional release helps individuals express and process suppressed feelings, thereby reducing psychological tension and emotional distress (13). Meaning construction allows experiences to be reorganized into coherent narratives, which strengthens the integration between emotion and cognition and promotes a stable sense of self (14). Cognitive reappraisal enables individuals to reinterpret stressful experiences from a more adaptive perspective, improving psychological flexibility and resilience (15). Together, these processes foster emotional regulation, self-awareness, and personal growth. For patients with schizophrenia, who often present with emotional flattening, fragmented self-concept, and disorganized thinking, EW provides a structured and accessible way to reconnect emotional experience with cognitive processing.

#### 2.1.2 Clinical applications in schizophrenia

Growing evidence supports the potential value of EW in the rehabilitation of schizophrenia. Randomized controlled studies have shown that writing interventions emphasizing positive psychological themes such as gratitude, hope, and personal meaning can enhance well-being, reduce self-stigma, and improve life satisfaction (16). Participants who engage in EW demonstrate greater emotional expression, reduced anxiety, and stronger self-reflection compared with those in neutral writing conditions (7). These effects are particularly relevant for individuals with impaired metacognitive ability and limited emotional awareness. By encouraging patients to construct coherent personal narratives, EW may strengthen insight, improve emotional connectedness, and promote engagement in psychosocial rehabilitation. From a cognitive perspective, the act of transforming inner experience into language may activate neural circuits related to attention, emotion regulation, and executive control, thereby complementing traditional therapeutic approaches that mainly target symptom reduction (17).

#### 2.1.3 Limitations and challenges

Despite promising findings, current research on EW in schizophrenia faces several limitations. Most studies are limited by small sample sizes, short intervention periods, and methodological heterogeneity, which restrict the reliability and generalizability of conclusions. Cultural and linguistic differences may also influence writing content and emotional depth, complicating the standardization of intervention protocols (18). Moreover, the majority of studies rely on subjective questionnaires, lacking objective or quantitative indicators to evaluate therapeutic effects. The absence of standardized outcome measures makes it difficult to clarify the underlying mechanisms and long-term benefits. In addition, variability in literacy, motivation, and symptom severity may reduce adherence to writing tasks and affect consistency of results.

Future studies should aim to combine EW with objective evaluation methods such as linguistic analysis or digital monitoring to capture changes in emotional and cognitive states more precisely. Establishing culturally sensitive, evidence-based frameworks will be essential to realize the clinical potential of EW as a low-cost and scalable psychosocial intervention for patients with schizophrenia.

### 2.2 Digital cognitive therapy in schizophrenia

#### 2.2.1 Definition and types

DCT refers to the application of technology-based platforms to deliver structured cognitive and behavioral interventions through computers, mobile devices, or virtual environments. It integrates principles from CBT, neurocognitive rehabilitation, and digital health design to enhance accessibility and engagement (19). Major forms of DCT include digitalized CBT, computer-assisted cognitive remediation training, and virtual reality (VR)-based therapeutic systems (20). Digital CBT uses interactive modules, multimedia materials, and real-time feedback to guide patients in identifying and restructuring maladaptive thoughts and behaviors (21). Computerized cognitive training focuses on improving specific cognitive domains such as attention, working memory, and executive function through repetitive and adaptive tasks. Virtual reality interventions create immersive environments that simulate real-life social interactions and daily scenarios, helping patients practice adaptive behavior, improve social cognition, and reduce anxiety in a safe and controlled setting (22). Collectively, these digital modalities allow for flexible, individualized, and data-driven approaches to cognitive and functional rehabilitation in schizophrenia.

#### 2.2.2 Clinical evidence and efficacy

A growing body of clinical research, including schizophrenia-specific randomized controlled trials and meta-analyses, supports the efficacy of computer-assisted cognitive remediation (CACR) in improving cognitive and functional outcomes in this population (23–25). For instance, a recent trial demonstrated that CACR significantly improved personal recovery, autonomy, competence, and engagement in meaningful activities in individuals with schizophrenia (24). Randomized controlled trials have demonstrated that computer-based cognitive training can lead to significant improvements in attention, memory, and executive functioning, which are often resistant to pharmacological therapy. These cognitive gains are associated with better social functioning, enhanced occupational performance, and reduced negative symptoms (26). Digital CBT interventions have also shown benefits in reducing depressive symptoms, mitigating paranoid ideation, and promoting insight and self-efficacy. Virtual reality–assisted therapies further contribute to social skills development, emotional recognition, and functional recovery by providing realistic yet safe contexts for behavioral rehearsal (27).

Meta-analyses have confirmed that DCT produces moderate to large effect sizes in cognitive and functional outcomes, particularly when combined with psychosocial rehabilitation or clinical supervision (28). Importantly, digital interventions have demonstrated feasibility across both inpatient and community settings, supporting their potential as scalable tools for long-term management and relapse prevention.

#### 2.2.3 Advantages and challenges

DCT offers significant advantages in the rehabilitation of schizophrenia by providing flexible, accessible, and cost-effective treatment options. Its structured and interactive design enables individualized cognitive training, immediate feedback, and objective progress monitoring, which together enhance treatment efficiency and scalability (29). The use of gamified tasks and multimedia elements further increases engagement and motivation, supporting sustained participation and potentially improving cognitive and functional outcomes (30). However, despite these benefits, several challenges hinder its optimal implementation. Poor adherence remains the most critical issue, as patients with schizophrenia often exhibit reduced motivation, cognitive fatigue, and limited technological familiarity. Barriers related to device availability, internet access, and privacy concerns also restrict widespread use. Moreover, automated digital systems may fail to capture the complexity of emotional and cognitive changes, leading to insufficient personalization of therapy (31). Thus, while DCT represents a promising and scalable approach to cognitive rehabilitation, its success ultimately depends on improving patient adherence, ensuring accessibility, and maintaining individualized therapeutic relevance.

### 2.3 Integrative mechanistic framework of EW + DCT

#### 2.3.1 Complementary mechanisms

EW and DCT represent two distinct yet complementary therapeutic approaches that address different aspects of emotional and cognitive dysfunction in schizophrenia (32). EW facilitates emotional processing, meaning construction, and cognitive reappraisal, helping patients articulate internal experiences and restore coherence between thoughts and emotions (33). This process not only enhances emotional regulation but also fosters self-awareness and intrinsic motivation for change. Evidence from recent schizophrenia-specific research supports these mechanisms. For example, a randomized controlled trial (RCT) with 54 hospitalized female patients with schizophrenia demonstrated that a structured, 2-week positive psychology expressive writing intervention significantly improved specific outcome domains—including reduced perceived stigma, and increased hope, adaptive coping style, and quality of life—compared to routine care (34). Furthermore, a case report indicates the feasibility of expressive writing for enhancing self-disclosure ability in clinical settings with patients experiencing hallucinations (35). In contrast, DCT focuses on structured cognitive training and behavioral activation through repetitive, goal-directed exercises that strengthen attention, memory, and executive control (36). When integrated, these two modalities form a synergistic mechanism in which EW prepares the emotional and motivational foundation for effective participation in cognitive training, while digital therapy consolidates these emotional gains through measurable cognitive improvement and behavioral adaptation.

#### 2.3.2 Integrative model and potential effects

The integration of EW and DCT creates a dual-pathway model targeting both emotional and cognitive domains of recovery. Writing tasks can activate self-reflection and emotional repair, thereby increasing intrinsic motivation and readiness for engagement in digital training sessions. This heightened motivation may enhance adherence to DCT and amplify its cognitive and functional benefits (37). However, at present, there is insufficient direct evidence of this synergistic effect in patients with schizophrenia. Meanwhile, digital cognitive exercises can strengthen cognitive flexibility and behavioral activation, which in turn reinforce the emotional insights and self-efficacy gained from writing practice (38). Through this two-way interaction, this comprehensive intervention program has the potential to alleviate negative symptoms, improve social functions and promote sustained recovery. Further validation is needed in clinical studies.

**Figure 1:**
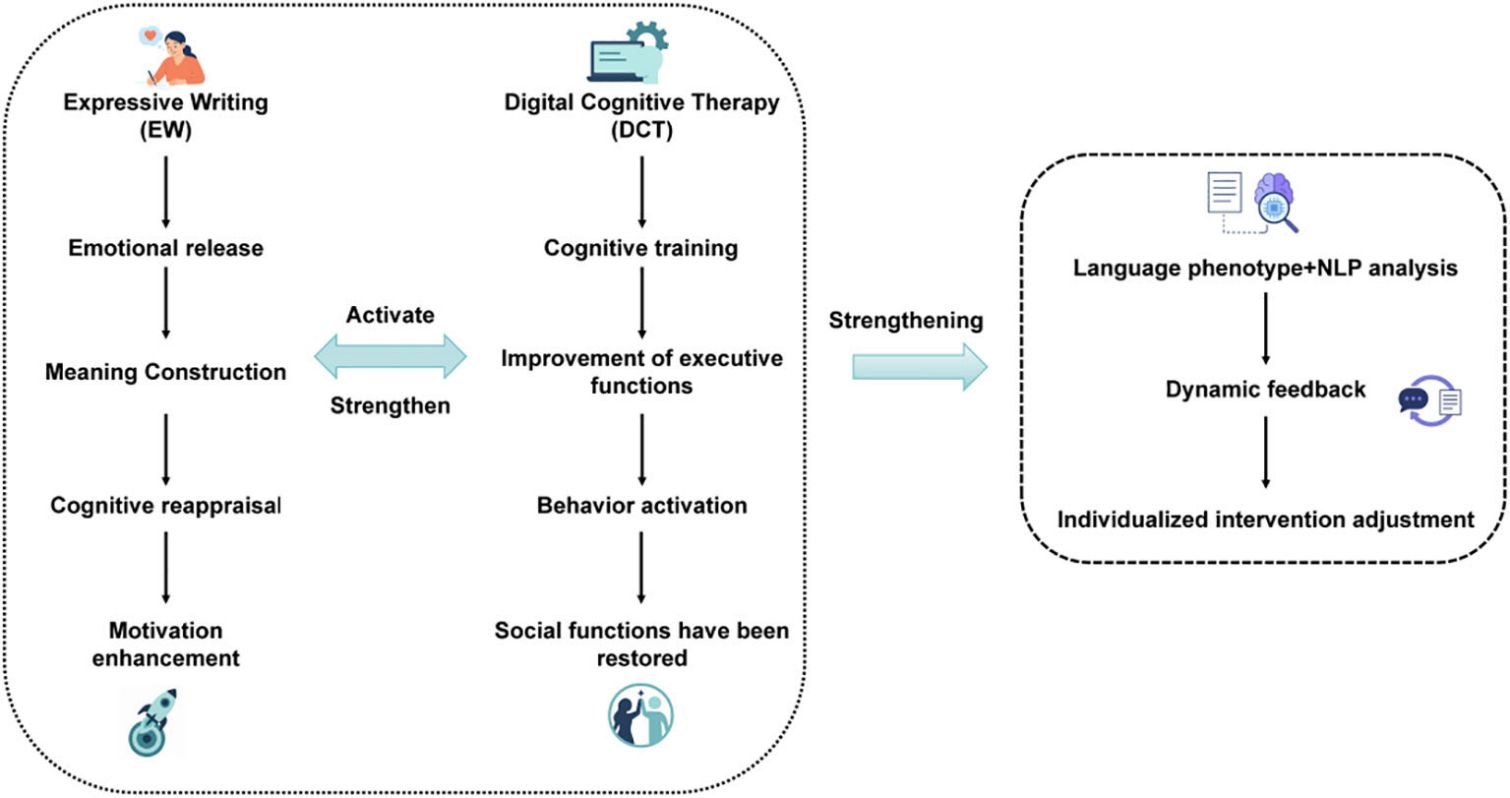
EW + DCT integrated model dynamic feedback mechanism.

It is important to note that this synergistic model is primarily theoretical and derived from extrapolating evidence from separate studies on EW and DCT in schizophrenia, as well as from other populations. Direct empirical evidence from studies evaluating the combined EW+DCT intervention in schizophrenia is currently lacking, which underscores the need for future research to test its proposed benefits. As illustrated in **Figure 1**, the EW + DCT integrative model operates through two interactive pathways: an emotional-cognitive channel that enhances emotional expression and meaning-making, and a cognitive-behavioral channel that supports learning, adaptation, and social reintegration. The dynamic feedback between these pathways contributes to a more comprehensive and enduring therapeutic effect.

#### 2.3.3 Practical applications

The combined EW + DCT intervention may be most suitable for outpatients in the rehabilitation phase or individuals experiencing early psychosis, who often retain sufficient cognitive capacity and insight to benefit from structured self-guided activities. The model can be implemented through online or mobile platforms that integrate EW modules with interactive cognitive training programs, forming a blended intervention system within mobile health (mHealth) frameworks. This digitalized delivery mode enables flexible scheduling, remote supervision, and continuous data monitoring, which together facilitate personalized treatment and long-term adherence. By merging emotional engagement with cognitive reinforcement, the EW + DCT approach may offer a promising research direction for scalable and integrative rehabilitation, but its feasibility and effectiveness in schizophrenia remain to be established.

### 2.4 Linguistic phenotypes and NLP-based evaluation

#### 2.4.1 Relationship between language abnormalities and schizophrenia

Language disturbance is a hallmark feature of schizophrenia and reflects the underlying disorganization of thought processes. Patients often exhibit disordered speech characterized by fragmented syntax, incoherence, and semantic drift, which mirror deficits in cognitive control and abstract reasoning (39). Reduced lexical diversity and impoverished semantic content are frequently observed, particularly in those with prominent negative symptoms (40, 41). These abnormalities not only impair communication but also provide insight into the cognitive and neural mechanisms that underlie the illness (42). Since language production is closely linked to cognitive function, analyzing linguistic behavior offers a valuable window into the integrity of thought, affect, and social cognition in schizophrenia.

#### 2.4.2 Applications of natural language processing in schizophrenia research

Advances in NLP have enabled the quantitative analysis of linguistic data in psychiatric research, providing objective and reproducible measures of thought and communication patterns (43). NLP-based methods can quantify semantic coherence, syntactic complexity, and lexical diversity, allowing for the detection of subtle deviations associated with thought disorder. Studies have shown that reduced semantic connectivity and increased linguistic disorganization are strongly correlated with the severity of formal thought disorder, negative symptoms, and impaired social functioning (44). Computational models have also demonstrated predictive value in identifying individuals at high risk for psychosis and in monitoring symptom progression over time (45). By transforming natural language into quantifiable markers, NLP contributes to a deeper understanding of schizophrenia as a disorder of language-mediated cognition.

#### 2.4.3 Potential applications of linguistic phenotypes in EW + DCT

In the integrated EW and DCT framework, linguistic phenotypes derived from NLP analysis can serve as objective indicators of emotional and cognitive changes during intervention (46). Features such as semantic richness, affective tone, and syntactic organization may reflect the degree of emotional engagement, cognitive coherence, and motivation elicited by EW. These language-derived metrics can be used to evaluate treatment response more precisely than self-reported questionnaires and provide real-time feedback on therapeutic progress. For example, increasing semantic coherence and emotional valence in writing samples may indicate improved cognitive integration and emotional regulation, while linguistic impoverishment or affective flattening could signal reduced engagement or emerging relapse risk (47). Integrating these dynamic linguistic markers into digital cognitive platforms allows continuous monitoring of patients’ psychological states and adherence patterns, supporting individualized and adaptive rehabilitation.

To translate these potential applications into a clinically actionable framework, several methodological steps are required. First, regarding robust measures, initial evidence points to semantic coherence and lexical diversity as particularly promising and quantifiable markers. These can be operationalized in standardized schizophrenia writing tasks using established NLP pipelines—for instance, applying Latent Semantic Analysis (LSA) to compute semantic coherence and calculating type-token ratio (TTR) for lexical diversity (48). Second, validation against clinical benchmarks is essential. These computational linguistic measures should be correlated with gold-standard symptom scales, including PANSS total, negative symptom, and disorganization scores, as well as functional outcome assessments such as SOFAS or PSP. Longitudinally, changes in linguistic markers before and after EW+DCT should be tested against changes in symptom severity, cognitive performance, adherence, and social functioning to determine whether they can serve as sensitive markers of therapeutic response (49). Finally, key confounding variables must be systematically addressed before linguistic phenotypes can be interpreted clinically. Future studies should control for years of education, literacy level, primary language or dialect, baseline verbal IQ, antipsychotic dose or chlorpromazine-equivalent dose, medication-related psychomotor slowing, illness duration, and symptom subtype. These factors can be handled through stratified recruitment, minimum literacy inclusion criteria, standardized language prompts, subgroup analyses, and multivariable models in which linguistic features are adjusted for demographic, clinical, and treatment-related covariates.

#### 2.4.4 Limitations and challenges

Despite its promise, the application of linguistic and NLP-based approaches in schizophrenia research and intervention faces several challenges. Cultural and linguistic diversity can influence language structure and expression, complicating cross-population comparability and algorithm generalization. Data privacy and ethical considerations are critical, as language data may contain sensitive personal information. Furthermore, the interpretability of computational models remains limited; algorithmic outputs often lack transparent links to clinical constructs, making it difficult for clinicians to integrate these findings into practice. To address these issues, future developments should emphasize culturally adapted corpora, transparent modeling frameworks, and robust data governance systems to ensure both validity and ethical integrity in the use of language-based evaluation tools.

Furthermore, the successful implementation of an integrated EW+DCT model hinges critically on careful patient selection and addressing practical feasibility barriers. The intervention may not be suitable or could even be harmful for certain patient subgroups. Individuals with severe formal thought disorder or active psychosis may find the structured demands of expressive writing and cognitive tasks overwhelming. Patients with very low literacy or limited education may struggle with the written component, leading to disengagement. High levels of paranoia or distrust towards digital technology—a not uncommon feature in schizophrenia—could result in non-adherence or distress. Finally, socioeconomic barriers and lack of reliable access to digital devices would systematically exclude a vulnerable population, potentially widening existing healthcare disparities. Therefore, future research and clinical translation must explicitly define inclusion and exclusion criteria, develop adapted protocols (e.g., simplified interfaces, voice-input options, in-person support), and consider strategies like providing loaned devices to ensure equitable and safe access.

### 2.5 Adherence modulators and optimization strategies

#### 2.5.1 Importance of adherence

Adherence is a critical determinant of treatment efficacy in digital interventions for schizophrenia. Numerous studies have demonstrated a clear dose–response relationship between the duration of DCT use and the magnitude of clinical improvement (50). Patients who maintain consistent engagement tend to exhibit greater cognitive gains, enhanced emotional regulation, and improved social functioning. Conversely, low adherence often leads to partial or transient benefits, undermining the long-term effectiveness of even well-designed digital programs (51). Therefore, understanding and enhancing adherence is essential to ensure that digital interventions achieve their intended therapeutic impact.

#### 2.5.2 Influencing factors

Multiple factors influence adherence to digital therapy among individuals with schizophrenia. Technical accessibility plays a foundational role, as reliable devices, stable internet connections, and user-friendly interfaces determine whether patients can participate consistently. Psychological factors such as self-efficacy and intrinsic motivation strongly affect engagement, with patients who perceive themselves as capable of self-directed learning showing higher persistence. Symptom-related variables also contribute: negative symptoms, cognitive fatigue, and disorganized thinking can reduce attention and motivation, while the severity of psychotic symptoms may disrupt continuity of use. Environmental and social contexts, including caregiver involvement and clinical support, further shape adherence behavior (52, 53). Recognizing these diverse determinants provides a framework for targeted strategies to improve engagement and retention. Furthermore, future research should systematically examine the role of demographic (e.g., gender, age) and clinical variables (e.g., duration of illness, symptom severity), as well as intervention setting (individual vs. group format), in moderating adherence and treatment outcomes.

#### 2.5.3 Optimization strategies

Enhancing adherence requires a multidimensional approach that combines motivational, technical, and behavioral strategies. Incorporating gamified elements and reward systems can transform repetitive cognitive exercises into enjoyable tasks, reinforcing motivation through positive feedback. Personalized reminders, adaptive difficulty adjustments, and progress visualization promote a sense of accomplishment and sustained participation. Peer support and clinician feedback within digital platforms can also foster accountability and social connection, which are particularly valuable for patients with limited interpersonal interaction. Moreover, integrating language and behavioral data enables dynamic monitoring of engagement patterns; automated detection of reduced participation or emotional withdrawal can trigger timely interventions, such as supportive messages or adjusted task complexity (54). Through these design principles, digital interventions can become more responsive, engaging, and patient-centered.

#### 2.5.4 Interaction with linguistic phenotypes

Language features extracted from EW or in-app communication can serve as sensitive markers of adherence trends. Artificial intelligence (AI) and NLP techniques are increasingly employed to automate the analysis of such text and voice data, enabling more precise and scalable assessment of these linguistic phenotypes. Subtle changes in linguistic coherence, emotional tone, or vocabulary diversity may precede behavioral signs of disengagement, offering an early signal of declining motivation (55). By combining linguistic indicators with usage data, clinicians can identify at-risk individuals and provide personalized support before significant deterioration occurs. This integration of linguistic monitoring and behavioral analytics establishes a feedback loop that enhances both adherence and treatment precision (56). Leveraging these dynamic interactions between language and engagement has the potential to transform DCT into a more adaptive and proactive model of rehabilitation for schizophrenia.

## 3 Future directions

### 3.1 Validation through multicenter randomized controlled trials

Future studies should prioritize the design of multicenter randomized controlled trials to validate the combined effectiveness of EW and DCT in schizophrenia rehabilitation. Large-scale clinical research is necessary to confirm the synergistic effects of emotional expression and cognitive training, while also identifying the patient populations that benefit most from this integrated approach. Standardized intervention protocols, objective cognitive and linguistic outcome measures, and long-term follow-up will be essential to determine the stability and generalizability of treatment effects across different clinical settings.

### 3.2 Development of AI-driven individualized intervention models

Building on the concept of linguistic phenotypes, future work should focus on developing artificial intelligence–based models capable of delivering individualized, adaptive interventions. By continuously analyzing patients’ language patterns, emotional tone, and engagement behaviors, these systems could identify changes in cognitive and emotional states and automatically adjust the content and intensity of interventions. The establishment of such AI-assisted feedback mechanisms would enhance precision, improve adherence, and allow dynamic optimization of rehabilitation strategies tailored to individual needs.

### 3.3 Localization and clinical translation of prescription digital therapeutics

Another key direction is the exploration of localized digital therapeutics for schizophrenia rehabilitation. The integration of EW and DCT into clinically approved, prescription-based digital tools could promote standardized and evidence-based digital treatment delivery. Localization efforts should address cultural adaptation, language customization, and healthcare infrastructure alignment to ensure that interventions are feasible and acceptable in diverse clinical environments. Strengthening clinician training and regulatory support will further accelerate the clinical translation of this model into everyday psychiatric care.

### 3.4 Ethical governance and interdisciplinary collaboration

The advancement of linguistically informed and AI-driven digital interventions must be accompanied by rigorous attention to data ethics and model transparency. Protecting patient privacy, ensuring informed consent, and maintaining algorithm interpretability are crucial to building trust and clinical credibility. Future research should also emphasize the establishment of requires interdisciplinary collaboration among clinicians, psychologists, data scientists, speech and language therapists, clinical linguists, and ethicists to guide responsible innovation. Constructing transparent clinical governance frameworks will help balance technological advancement with ethical integrity, supporting the sustainable integration of digital mental health solutions into clinical practice.

## 4 Conclusion

The integration of EW and DCT may provide a conceptual approach to the rehabilitation of schizophrenia by addressing both emotional and cognitive dimensions of recovery. EW promotes emotional processing and meaning construction, while DCT enhances cognitive flexibility and behavioral activation. These components collectively form an interactive framework, which is expected to alleviate negative symptoms, enhance cognitive functions, and improve social functions. The inclusion of linguistic phenotypes and adherence data further enables objective assessment and continuous adjustment of interventions, advancing the precision and individualization of treatment. Overall, this integrated model represents a proposed theoretical framework that could offer a potentially feasible and scalable strategy for promoting sustained rehabilitation and long-term management in schizophrenia. It is important to emphasize that this represents a hypothesis for future testing; direct clinical trial evidence for the combined intervention in schizophrenia is not yet available. Therefore, its clinical translation and effectiveness remain to be established through future empirical validation.
